# Identification of a novel selective PPARγ ligand with a unique binding mode and improved therapeutic profile *in vitro*

**DOI:** 10.1038/srep41487

**Published:** 2017-01-27

**Authors:** Wei Yi, Jingjing Shi, Guanguan Zhao, X. Edward Zhou, Kelly Suino-Powell, Karsten Melcher, H. Eric Xu

**Affiliations:** 1 School of Pharmaceutical Sciences, Guangzhou Medical University, Xinzao, Panyu District, Guangzhou 511436, P. R. China; 2 VARI/SIMM Center, Center for Structure and Function of Drug Targets, CAS-Key Laboratory of Receptor Research, Shanghai Institute of Materia Medica, Chinese Academy of Sciences, Shanghai 201203, P. R. China; 3 Laboratory of Structural Sciences, Program of Structural Biology and Drug Discovery, Van Andel Research Institute, Grand Rapids, Michigan 49503, USA

## Abstract

Thiazolidinediones (TZD) function as potent anti-diabetic drugs through their direct action on the nuclear receptor peroxisome proliferator-activated receptor γ (PPARγ), but their therapeutic benefits are compromised by severe side effects. To address this concern, here we developed a potent “hit” compound, VSP-51, which is a novel selective PPARγ-modulating ligand with improved therapeutic profiles *in vitro* compared to the multi-billion dollar TZD drug rosiglitazone (Rosi). Unlike Rosi, VSP-51 is a partial agonist of PPARγ with improved insulin sensitivity due to its ability to bind PPARγ with high affinity without stimulating adipocyte differentiation and the expression of adipogenesis-related genes. We have determined the crystal structure of the PPARγ ligand-binding domain (LBD) in complex with VSP-51, which revealed a unique mode of binding for VSP-51 and provides the molecular basis for the discrimination between VSP-51 from TZDs and other ligands such as telmisartan, SR1663 and SR1664. Taken together, our findings demonstrate that: a) VSP-51 can serve as a promising candidate for anti-diabetic drug discovery; and b) provide a rational basis for the development of future pharmacological agents targeting PPARγ with advantages over current TZD drugs.

PPARγ is a master regulator of adipose cell differentiation and development^1^,^2^,^3^,^4^. Structurally, PPARγ belongs to the nuclear hormone receptor superfamily. It is also well known as the target protein for the TZD class of anti-diabetic drugs such as Rosi (Avandia) and pioglitazone (Pio, Actos)^5^,^6^,^7^,^8^,^9^,^10^. From a clinical perspective, these TZD drugs act as full agonists of PPARγ and are highly effective oral medications for the treatment of type 2 diabetes mellitus (T2DM). However, many clinical studies have shown that administration of these TZD drugs is associated with a number of undesirable side effects, such as obesity, fluid retention, weight gain, cardiac hypertrophy, hepatotoxicity, and loss of bone mineral density^11^,^12^,^13^,^14^,^15^. For example, Avandia has been withdrawn from the European market and has also been restricted by the FDA due to increased cardiovascular risks associated with its use. More recently, Actos has also been found to have many controversial side effects, including the increased risk for bladder cancer^16^. Considering the increasing global epidemic of T2DM, undoubtedly, there is an urgent need to search and develop novel PPARγ-targeted anti-diabetic drugs with improved therapeutic profiles.

The pharmacological actions of PPARγ agonists are mediated through the PPARγ LBD, which includes the transcriptional AF-2 motif associated with helix 12 of the LBD^17^,^18^,^19^. Many structural and biochemical studies have demonstrated that the flexible AF-2 motif plays a critical role in the regulation of PPARγ-targeted genes^20^,^21^,^22^,^23^,^24^,^25^,^26^, thereby helping to elucidate the mechanism of ligand-induced transcriptional activation by PPARγ. In the absence of any ligand, the AF-2 helix is in equilibrium between closed (active) and open (inactive) conformations^27^. Upon binding of an activating full agonist, the AF-2 helix is locked in an active conformation, which allows the recruitment of co-activators required for transcriptional activation^28^. Indeed, the TZD class of drugs exhibited their efficient anti-diabetic effects via an AF-2-mediated “lock” mechanism. However, several studies have revealed that the full agonism associated with strong PPARγ transcriptional activities and “locking” of the AF2 helix in the closed conformation is also responsible for the TZD side effects^29^,^30^,^31^. A recently recognized challenge has therefore been the development of unique PPARγ ligands that stabilize the AF-2 helix in distinct states between closed and open conformations to selectively recruit co-activators that are associated with therapeutic benefits with reduced side effects^32^,^33^,^34^.

Over the last few years a large number of naturally occurring and synthetic non-TZD classes of novel PPARγ agonists or partial agonists have been developed as potential anti-diabetic drugs. Among them, the so called “Selective PPARγ modulators (SPPARγMs)” have attracted considerable attention because of their ability to selectively target PPARγ activity states^35^,^36^,^37^,^38^,^39^,^40^,^41^. In theory, these compounds are suspected to display PPARγ binding modes that are different from those of full agonists, including the presence of multiple receptor populations in intermediate conformational states, the conformational exchange within the ligand binding pocket and AF-2 region, and the specific receptor stabilization by binding to different types of ligands^33^. Thus, these compounds should have the ability to specifically target and activate selective co-activators to deliver therapeutic efficacy with minimized unwanted side effects. However, up to now, no anti-diabetic SPPARγMs have been successfully used in clinical practice and mechanistically it remains unclear how to achieve selective PPARγ activation.

Here we report a new and alternative PPARγ ligand with a desirable profile in the regulation of PPARγ, and then reveal its PPARγ LBD binding mode. In addition to the development of this specific novel PPARγ ligand with improved therapeutic profiles *in vitro*, this study also provides a molecular framework for future developments of pharmacological PPARγ agonists with advantages over current TZD drugs.

## Results

### The synthesis of novel indoles

**Figure 1:**
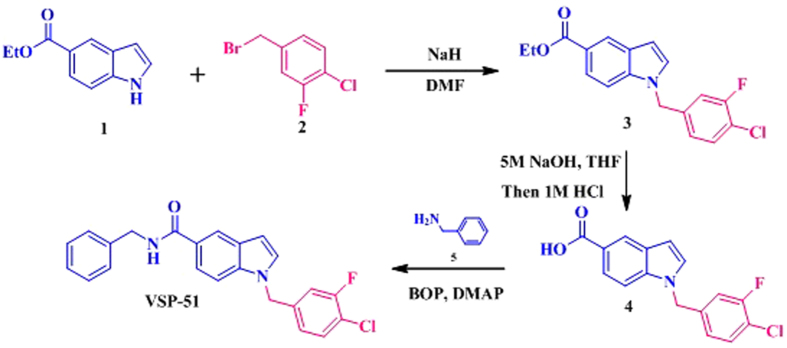
The synthesis of VSP-51.

With the reported PPARγ partial agonists benzimidazoles (scaffold 1)^42^ and indoles (scaffold 2)^43^ as the leads, four novel indoles VSP-19, VSP-25, VSP-51 and VSP-62 (scaffold 3) were designed and synthesized (Fig. S1). These indoles were routinely prepared in three synthetic steps from commercially available starting materials. Taken VSP-51 for example, as shown in Fig. 1, starting from ethyl 1H-indole-5-carboxylate **1** and 4-(bromomethyl)-1-chloro-2-fluorobenzene **2**, N-benzyl substituted indole **3** was synthesized via the classical SN_1_ alkylation reaction. Hydrolysis and subsequent amidation with phenylmethanamine **5** under standard coupling conditions gave the desired product VSP-51 in good yield.

### Identification of VSP-51 as a potent PPARγ ligand

**Figure 2:**
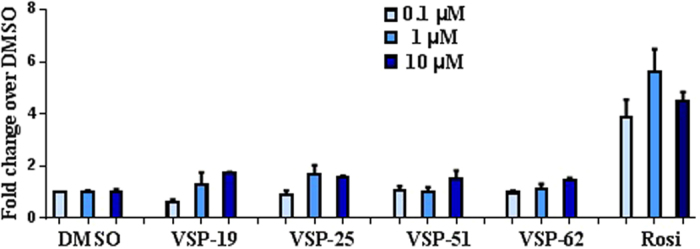
PPARγ luciferase reporter assay. Cos-7 cells were cotransfected with a PPARγ expression plasmid, a firefly luciferase gene under the control of three tandem PPARγ response elements, and a Renilla luciferase control. The firefly luciferase activity was normalized against Renilla luciferase units. Fold activation was calculated against DMSO vehicle. Fold activation of Rosi and compounds were at 0.1, 1 and 10 μM concentrations (n = 3, error bars = SEM).

With the synthetic compounds in hand, we first evaluated their agonistic activities for the human PPARγ subtypes by using a luciferase reporter assay in Cos-7 cells at 1 and 10 μM compound concentrations. Rosi, as a classical full PPARγ agonist, was used as a positive control. As summarized in Fig. 2, the results showed that all test compounds at the selected concentrations exhibited very weak PPARγ agonistic activities, which is similar to the negative control DMSO.

**Figure 3:**
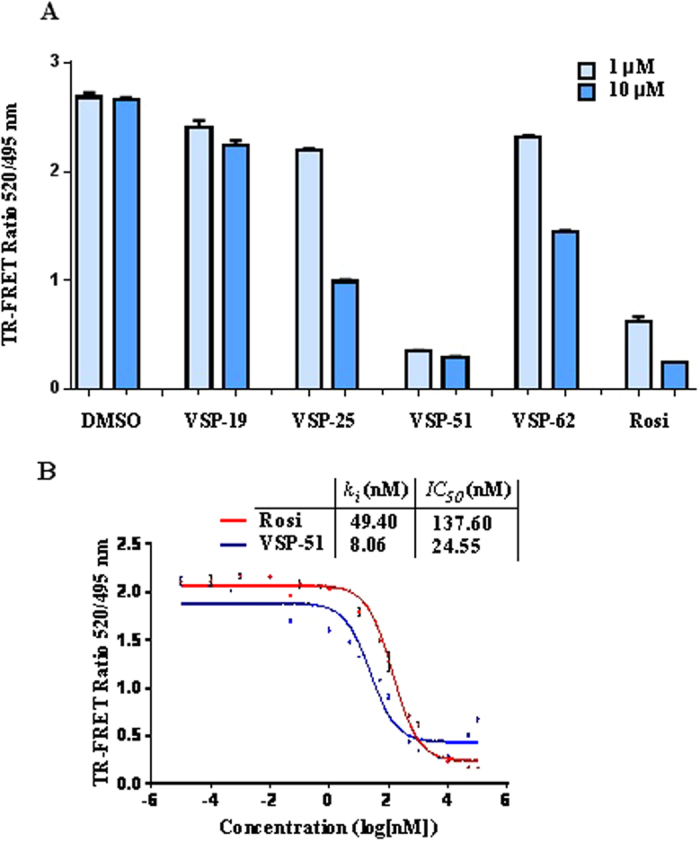
Characterization of VSP-51 binding. LanthaScreen TR-FRET assay (Invitrogen) was used for determining the relative binding affinities of the ligands VSP-19, 25, 51, 62 and Rosi. GST-PPARγ LBD and the fluorescent PPARγ ligand Fluormone™ were used at 0.5 nM of concentration, each. Terbium-coated anti-GST antibody was used at 4.95 nM of concentration. (**A**) LanthaScreen TR-FRET ratios for ligands at 1 and 10 μM of concentrations (n = 2, error bars = SEM). (**B**) Dose-response competition curves VSP-51 and Rosi in the range from 0.01 pM - 100 μM. Concentrations are presented on a log10 scale (n = 2, error bars = SEM).

To determine the PPARγ binding affinities of the synthesized compounds, we performed competition experiments using LanthaScreen TR-FRET assays. Figure 3A shows the reduction of the TR-FRET emission ratio, which is a measure for relative PPARγ affinities (the ability of compounds to compete binding of a fluorescently labeled PPARγ ligand) in the presence of 1 and 10 μM compounds, with Rosi and DMSO as the positive and negative control, respectively. As shown in Fig. 3A, VSP-51 has the highest binding affinity for PPARγ among the synthesized compounds. Very interestingly, the *IC*_*50*_ and *k*_*i*_ values of VSP-51 (*IC*_*50*_ = 22.45 nM, *k*_*i*_ = 8.06 nM) and Rosi (*IC*_*50*_ = 137.60 nM, *k*_*i*_ = 49.40 nM) indicate that VSP-51 has a more potent binding activity than Rosi (Fig. 3B). In combination with the data for transcriptional activation, we identified that VSP-51 is a potent modulating ligand with partial agonism towards PPARγ. This provides a basis for further evaluation of the novel VSP-51 as a potent candidate for anti-diabetic drug discovery. Thus, we focused on VSP-51 in our studies below.

### VSP-51 only marginally induces adipocyte differentiation and stimulates the expression of key adipogenic genes

**Figure 4:**
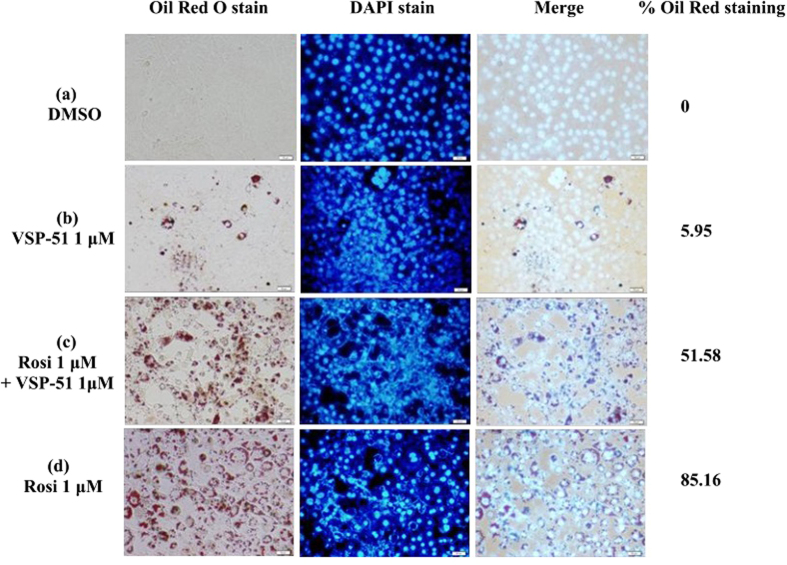
Lipid accumulation in differentiated 3T3-L1 cells treated with Rosi or VSP-51 following Oil Red O staining. 3T3-L1 fibroblast cells were induced by treatment with 1 μM of dexamethasone, 0.5 mM of isobutylmethylxanthine, and 850 nM of insulin for 48 h and cells were switched to maintenance medium containing 850 nM of insulin for 6 days.

One of the best documented side-effects of PPARγ agonistic drugs is weight gain as PPARγ is the key activator of adipogenesis. Therefore, we selected VSP-51 and tested its ability to promote differentiation of murine fibroblast 3T3-L1 cells to adipocytes, as monitored by the increase in Oil-red O staining. As shown in Fig. 4, Rosi potently stimulated adipocyte differentiation at the concentration of 1 μM as indicated by intensive Oil-red O staining of cytoplasmic fat droplets. In the control experiment, hardly any red cells were visible in the untreated cells. Interestingly, when 3T3-L1 cells were treated with 1 μM of VSP-51, very few cells were stained. Moreover, when VSP-51 was added during Rosi treatment, we observed a clear decrease in the total number of adipocytes. These results show that VSP-51 only marginally activates adipogenesis despite activating PPARγ, and moreover that VSP-51 can inhibit adipogenesis activated by Rosi, suggesting that it has reduced side effects compared to TZD drugs.

**Figure 5:**
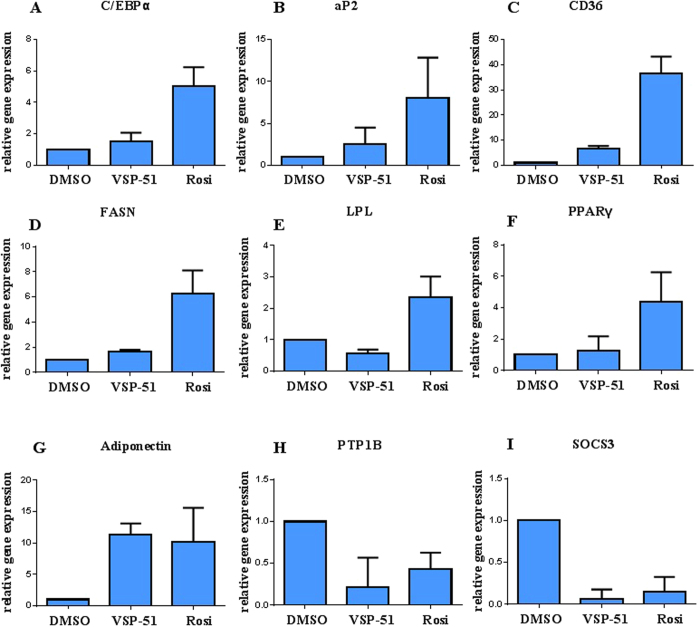
Expression of adipocyte-enriched (**A**–**F**) and insulin sensitivity-related (**G**–**I**) genes in 3T3-L1 cells was analyzed by quantitative PCR (qPCR). Relative mRNA levels of the adipocyte differentiation genes C/EBPα (adipogenic transcription factor CCAAT-enhancer-binding protein α), aP2 (fatty acid carrier adipocyte Protein 2), CD36 (fatty acid translocase cluster of differentiation 36), FASN (fatty acid synthase), and LPL (lipoprotein lipase), as well as PTP1B (protein tyrosine phosphatase 1B), SOCS3 (suppressor of cytokine signaling 3), and Adiponectin mRNA extracted from differentiating cells after 7 days (n = 3, error bars = SEM).

We further characterized the ability of VSP-51 to stimulate PPARγ activity by measuring the mRNA levels of endogenous PPARγ-regulated downstream genes linked to adipogenesis and insulin sensitivity (primers are listed in Table S1). Quantitative PCR was performed on 3T3-L1 cells treated with either VSP-51 or Rosi after 7 days. As shown in Fig. 5, Rosi robustly stimulated the expression of key adipogenic genes encoding C/EBPα (Fig. 5A), aP2 (Fig. 5B), CD36 (Fig. 5C), FASN (Fig. 5D), LPL (Fig. 5E) and PPARγ (Fig. 5F). In contrast, VSP-51 induced little or no change in the expression of these genes. The results further verify that VSP-51 is a safer PPARγ ligand than Rosi, which is also in good agreement with the above results from the Oil-red O staining assay. In contrast to adipogenic genes, VSP-51 efficiently increased the expression level of Adiponectin (Fig. 5G) and also remarkably inhibited the expression of PTPIB (Fig. 5H) and SOCS3 (Fig. 5I), revealing that VSP-51 contributes to improved insulin sensitivity. Taken together, these results provide evidence that VSP-51 has a potential advantage over TZD drugs for the treatment of T2DM.

### VSP-51 has a unique PPARγ binding mode

**Figure 6:**
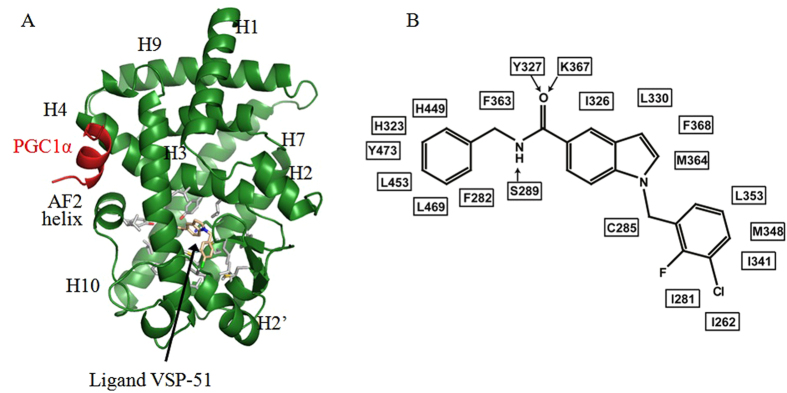
Crystal structure of PPARγ LBD in complex with VSP-51. (**A**) The overall PPARγ LBD structure (green) in complex with compound VSP-51 (pink) and a PGC1α peptide (red). Labeled are major secondary structure features, and the compound. The residues surrounding the compound are presented as sticks with carbons in grey, nitrogens in blue, and oxygens in red. (**B**) A schematic presentation of the interaction network between compound VSP-51 and the pocket residues of PPARγ. Arrows indicate H-bonds.

**Figure 7:**
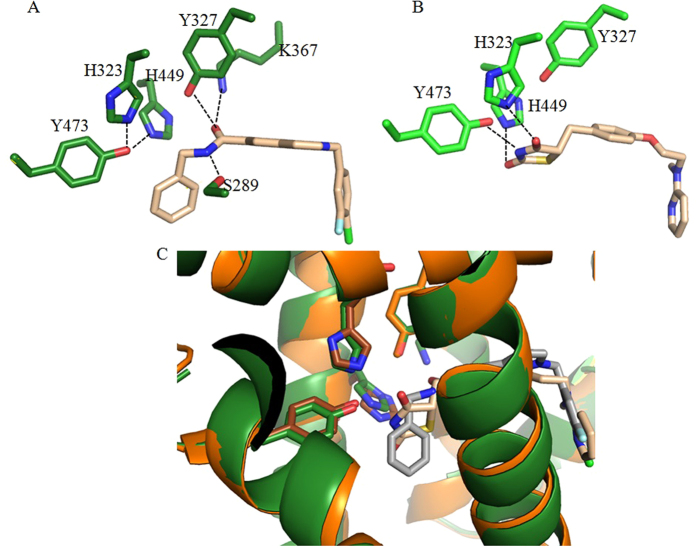
The PPARγ hydrogen bonding networks for VSP-51 (**A**) and Rosi (**B**). (**C**) Superposition of PPARγ structures in complex with VSP-51 (dark green) and Rosi (brown).

As a selective modulating ligand for PPARγ with partial agonism, VSP-51 has shown a distinct biological profile compared to TZDs. We therefore wanted to understand the structural basis for the recognition of this unique ligand by PPARγ. To reveal the PPARγ binding characteristics of VSP-51, we solved the crystal structure of PPARγ complexed with compound VSP-51 and a 15 amino acid LXXLL motif peptide of the PPARγ co-activator PGC1α1 by X-ray crystallography to a resolution of 1.93 Å (PBD code: 5TWO, Fig. 6A and Table 1). The compound VSP-51-binding pocket of PPARγ is enclosed by residues from helices 3, 4, 5, 6, 7, and 10 and the AF-2 helix as well as residues from the β strand and a loop region between helices 6 and 7. The majority of the pocket residues are hydrophobic residues (Fig. 6B), similar to the pocket of PPARγ bound to Rosi and other agonists^28^. The most specific feature of VSP-51 in complex with the PPARγ binding pocket is the hydrogen bonding network among the amide group of VSP-51 and the PPARγ residues Ser289 from helix 3, Tyr327 from helix 5, and Lys367 from helix 7 (Figs 6B and 7A). For comparison, the Rosi-PPARγ complex forms a different hydrogen binding system in which the amine and two carbonyl groups from the TZD head form hydrogen bonds with side chains of Tyr473, His323, and His449 of PPARγ, respectively (Fig. 7B). Notably, the amide group of VSP-51 does not interact with those three key residues, His323 at helix 5, His449 at helix 10, and Tyr473 from the AF-2 helix whose interaction with the acidic head groups of TZDs may contribute to the conformation of the receptor related to adverse effects of the TZD drugs. These interactions between VSP-51 and PPARγ also induce a slight change of the side chain rotamers of some receptor residues compared to those in the PPARγ-Rosi complex structure. This is especially the case for side chain groups that directly bind to ligands, including the gamma oxygen of Ser289 that forms a hydrogen bond with the amide nitrogen of VSP-51, and for His323 and His449, which move closer to and form hydrogen bonds with Tyr473 (Figs 7B and C) as also seen in the apo PPARγ structure^27^ (PBD code: 1PRG).

Next we compared the PPARγ/VSP-51 structure to structures of PPARγ in complex with chemically related PPARγMs for which structural information is available. Telmisartan, an angiotensin II receptor antagonist, plays roles as a PPARγ partial agonist that modulates the expression of PPARγ target genes involved in carbohydrate and lipid metabolism^44^. Telmisartan has a benzimidazole core instead of the central indole group in VSP-51. The crystal structure of the PPARγ LBD-telmisartan complex revealed a ligand-receptor binding mode different from that of VPS-51 (Fig. S2A), with the N3’ atom of the central benzimidazole ring forming a hydrogen bond with the receptor residue Tyr473 from helix 12, and the N1’ atom interacting with the gamma oxygen of Ser289 from helix 3 (Fig. S2B)^45^. The bulky propyl moiety and the terminal benzimidazole group of telmisartan pushed away the PPARγ side chains of His323 and Phe363 from their positions displayed in the PPARγ-VSP-51 complex (Fig. S2B vs Fig. S2C).

SR1663 and SR1664 are PPARγ ligands with C5-amide substituted indole moieties that are very similar to that of VSP-51 and whose amide groups also interact with Tyr327 of the protein. As reported, SR1664 is an antagonist that is similarly efficacious at insulin sensitizing as Rosi^46^. In contrast, SR1663, the enantiomer of SR1664, is an agonist of PPARγ that lacks the potent pharmacological activity demonstrated for SR1664^47^. The crystal structures of PPARγ in complexes with SR1663 and SR1664 show that both ligands partially overlap, and the amide groups of both compounds form hydrogen bonds with Tyr327, but the details of the bonds differ (Fig. S3). Specifically, SR1664 forms a hydrogen bond with the side chain of Tyr327 through its amide nitrogen (Fig. S3B), while SR1663 forms hydrogen bond with that residue through the carbonyl oxygen of its amide group (Fig. S3C). In addition, Tyr473 of the receptor, is too far away from the alkyl group (-CHCH3) of SR1664 for bond formation, but close enough to this group in the SR1663 complex to form a Van der Walls interaction (Fig. S3A–C) similar to that of the -CH2- group of VSP-51 (Fig. S3D and E).

It is interesting that VSP-51, which displays beneficial pharmaceutical activity comparable to that of SR1664, shares a PPARγ hydrogen bonding mode with the pharmacologically inactive SR1663 (Fig. S3C–E). This indicates that the hydrogen bonding between the amide group of the ligand and Tyr327 of the protein as well as the Van der Waals interaction between the alkyl group and Tyr473 is important, but not the only determining factor, for the therapeutic efficacy of these compounds.

**Figure 8:**
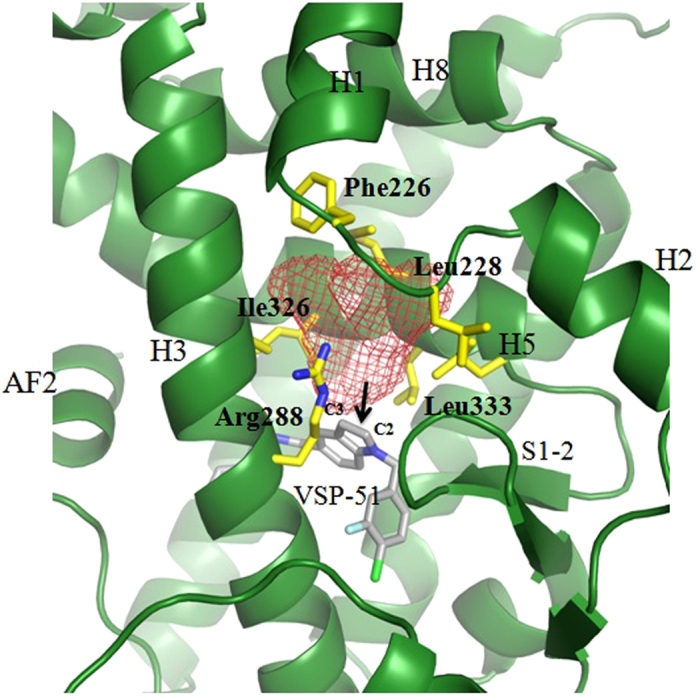
Structure-based discovery of C2 or/and C3-positions of the indole nucleus as a new starting point for future development of new pharmacophores selectively targeting PPARγ. The mesh indicates the available pocket space near the C2 and C3 positions of the indole core in the ligand.

In combination with these reported structural data, the conformational changes of backbone and side chains induced by VSP-51 indicate that the large LBD of PPARγ has great flexibility to adapt to the binding of diverse ligands. Notably, we also identified an inducible hydrophobic pocket in the VSP-51 complex crystal structure around the C2- and C3-positions of the indole nucleus, surrounded by Arg288 from helix 3, Ile326 and Leu333 from helix 5, and distantly Phe226, and Leu228 from the loop between helices 1 and 2 (Fig. 8), which also occurs in other PPARγ-ligand complex structures^47^,^48^. The existence of this pocket indicates that introducing a proper side group into the indole nucleus of our compound at C2- or/and C3- positions may be a new direction to modify the compound to improve its therapeutic efficacy targeting PPARγ.

## Discussion

Since the treatment of T2DM with TZD drugs is associated with many severe side effects, the development of non-TZD classes of new and alternative PPARγ ligands has attracted considerable attention in modern medicinal chemistry. In view of these developments, SPPARγMs with partial agonism in transcriptional activity were found to be advantageous not only as promising candidates for the treatment of T2DM but also as useful chemical probes for the elucidation of the biological function and regulation mechanism of PPARγ. In this paper, we identified a novel synthetic compound, VSP-51, which acts as a selectively modulating ligand for PPARγ. The results from both the biochemical LanthaScreen TR-FRET assay and the cell-based reporter gene assay demonstrate that VSP-51 is a novel partial agonist towards PPARγ. Compared with the typical full agonist Rosi, VSP-51 retains potent binding affinity for PPARγ but is compromised in its ability to activate the expression of adipogenic genes.

VSP-51 has several key features that distinguish it from TZDs. First, the scaffold of VSP-51 is distinct from that of TZDs, with the biologically important indole nucleus and amide moiety as two key structural motifs, which indicates an alternative drug design strategy for targeting PPARγ. Second, VSP-51 has only marginal adipogenic activity. A significant consequence of full agonist activation of PPARγ is the induction of adipocyte differentiation. PPARγ agonists, including marketed TZD drugs, have strong adipogenic potency and this is the major factor leading to their undesirable side effects. The results from the adipocyte differentiation experiments demonstrate that VSP-51 in contrast has almost no adipogenic activity, suggesting that VSP-51 has a potential advantage over TZD drugs. This conclusion was further supported by the expression analysis of PPARγ-regulated key adipogenic genes. Moreover, the preliminary anti-diabetic effect of VSP-51 has been confirmed by the expression analysis of insulin sensitivity-related key genes. Taken together, we have provided clear evidence that VSP-51 is a partial agonist for PPARγ that functions as a selectively PPARγ-modulating ligand with improved therapeutic profile compared to Rosi, suggesting that VSP-51 can serve as a promising candidate for the treatment of T2DM and as lead compound for designing newer and better pharmacophores selectively targeting PPARγ.

Evidence from our structural study reveals that VSP-51 has a unique mode of binding to the PPARγ LBD that is clearly distinct from that of TZDs. Rosi represents a topology typical for full PPARγ agonists, i.e., its head group forms a series of hydrogen bonds with the AF-2 helix while its tail occupies the large cavity of the binding site. Especially the strong hydrogen bonding interactions with the three key amino acid residues, His323 of helix 5, His449 of helix 10, and Tyr473 of the AF-2 helix is believed to be crucial to induce the recruitment of co-activators. However, these interactions are likely also associated with many adverse effects as mentioned by various reports. As revealed in the complex structure reported here, VSP-51 does not form hydrogen bonding interactions with the AF-2 helix, which may partly explain why VSP-51 functions as PPARγ partial agonist instead of full agonist. Instead, the amide group of VSP-51 interacts with the PPARγ residues Ser289 of helix 3, Tyr327 of helix 5, and Lys367 of helix 7. Compared to the PPARγ-Rosi complex structure, the receptor-VSP-51 crystal structure displays slight, but significant, shifts of protein backbone and side chain rotamers of the ligand binding pocket residues, including those of Tyr473, His323 and His449 that make them move toward and form hydrogen bonding between each other as occurs in the apo PPARγ structure. The unique binding characteristics of VSP-51 may reveal the molecular mechanism for selectively modulating PPARγ with distinctive properties, thereby providing a molecular basis for the discrimination of VSP-51 from TZD drugs and other ligands. Moreover, it reveals a new scaffold for the design of unique PPARγ ligands that retain potent anti-diabetic potencies, yet have reduced side effects.

In summary, here we have identified the synthetic compound VSP-51 as a novel PPARγ ligand. Compared to the currently marketed anti-diabetic drug Rosi, VSP-51 has several remarkable features: (i) VSP-51 is a selectively modulating ligand of PPARγ with a partial agonism; (ii) VSP-51 displays higher binding affinity for PPARγ than Rosi; (iii) VSP-51 only marginally induces adipocyte differentiation and does not or only marginally stimulate the expression of key fat cell genes, including C/EBPa, aP2, CD36, FASN and LPL; (iv) VSP-51 potently improves insulin sensitivity through selectively increasing the expression of Adiponectin and decreasing the expression of PTPIB and SOCS3; (v) VSP-51 has a unique mode of binding to PPARγ. More comprehensive clinical assays will be required to determine T2DM-related pharmacological and physiological actions *in vivo* to provide a comprehensive view on the anti-diabetic efficiencies *vs* adverse effects of VSP-51. In addition, tests will also be required to determine whether VSP-51 can block the phosphorylation of PPARγ at Ser273 by Cdk5, since the pioneering work by the research groups of Spiegelman and Griffin has uncovered that the inhibition of this phosphorylation tightly correlates with the anti-diabetic effects of PPARγ ligands^43^. Nevertheless, our results indicate that VSP-51 can serve as a promising candidate for effective and safer anti-diabetic drug discovery and as a lead compound for the development of new pharmacophores selectively targeting PPARγ. Moreover, the crystal structure of PPARγ bound to VSP-51 revealed an inducible PPARγ LBD pocket around the C2- and C3-positions of the indole nucleus that can be exploited for redesigning the compound to improve therapeutic efficacy as a potential anti-diabetic drug.

## Methods

### Procedure for the synthesis of VSP-51

(i) NaH (60% dispersion in mineral oil, 11.0 mmol) was added in portions at 0 °C to a stirred solution of ethyl 1*H*-indole-5-carboxylate **1** (1.89 g, 10.0 mmol) in dry DMF (10 mL). After stirring for 30 min at 0 °C, 4-(bromomethyl)−1-chloro-2-fluorobenzene **2** (2.65 g, 12.0 mmol) was added and then the mixture was stirred at room temperature for 24 h. Then, the reaction mixture was cooled to ambient temperature, poured into H_2_O (100 mL) and extracted with EtOAc (100 mL). The organic phase was dried over anhydrous Na_2_SO_4_. After evaporation of the solvents under reduced pressure, the crude product was purified on a silica gel column using EtOAc/petroleum ether (1:3) to get the pure **3** (2.80 g, 85%) as a white solid. (ii) A suspension of **3** (1.65 g, 5.0 mmol) and 5 M of NaOH solution (5 mL) in THF (20 mL) was stirred at 50 °C until consumption of the starting material. It was allowed to reach room temperature, 2 M of HCl (20 mL) was added, diluted with EtOAc (50 mL) and washed brine. The combined organic phase was dried over anhydrous Na_2_SO_4_. After evaporation of the solvents under reduced pressure, the crude product was purified by Flash chromatography to give the product **4** (1.35 g, 89%) as a white solid. (iii) To a solution of **4** (1.50 g, 5.0 mmol) in dry DMF (20 mL) were added 2.0 equiv of DMAP (1.22 g, 10 mmol) and 2 equiv of BOP (4.42 g, 10 mmol) at 0 °C in an ice-bath. After stirring for 0.5 h, 1.2 equiv of phenylmethanamine **5** (0.65 g, 6.0 mmol) was added, and the reaction was allowed to stir until all starting material had disappeared. Then, the mixture was poured into a 10% aqueous solution of HCl (50 mL). The aqueous phase was extracted with EtOAc (2 times), and the combined organic layers were dried (anhydrous Na_2_SO_4_) and concentrated in vacuo. Flash chromatography (Silica gel, hexane/ethyl acetate = 3/1) gave the desired product VSP-51 (1.66 g, 83%) as a colorless solid. The other three designed PPARγ ligands VSP-19, VSP-25 and VSP-62 were synthesized by using the same procedure.

### Characterization of VSP-51

^1^H NMR (400 MHz, DMSO-*d*_6_) *δ*: 8.92 (s, 1 H), 8.19 (s, 1 H), 7.75–7.65 (m, 1 H), 7.62 (d, 1 H, *J* = 3.2 Hz), 7.56–7.48 (m, 2 H), 7.32 (s, 2 H), 7.31 (s, 2 H), 7.27 (dd, 1 H, *J* = 10.3 and 1.8 Hz), 7.24–7.20 (m, 1 H), 7.00 (dd, 1 H, *J* = 8.3 and 1.4 Hz), 6.62 (d, 1 H, *J* = 3.1 Hz), 5.49 (s, 2 H), 4.49 (d, 2 H, *J* = 6.0 Hz);^13^C NMR (125 MHz, DMSO-*d*_6_) *δ*: 167.01, 157.08 (d, *J* = 247.2 Hz), 140.08, 139.86 (d, *J* = 6.4 Hz), 137.09, 130.82, 130.42, 128.20, 127.72, 127.12, 126.58, 125.91, 124.20 (d, *J* = 3.5 Hz), 120.88, 120.46, 118.35 (d, *J* = 17.4 Hz), 115.47 (d, *J* = 21.3 Hz), 109.66, 102.55, 48.11, 42.57;HRMS (ESI) m/z calculated for [M + H]^+^: 393.1170, found: 393.1165.

### Protein preparation

The LBD of PPARγ (codons 206–477) was cloned into the pSUMO vector (LifeSensors) with an N-terminal 6 × His SUMO tag. The protein was expressed in *E. coli* BL21 (DE3) in LB broth at 25 °C to an A_600_ of 1.0 and induced with 0.1 mM of isopropyl 1-thio-β-d-galactopyranoside at 16 °C. Cells were harvested, resuspended in 200 ml of extract buffer (50 mM of Tris (pH 8.0), 150 mM of NaCl, 10% of glycerol, and 25 mM of imidazole) per 6 liters of cells, and passed three times through a French press with pressure set at 1000 Pa. The lysate was cleared by centrifugation at 48,000× g for 30 minutes and the supernatant was purified by loading on 2 × 5 mL HiLoad Nickel HP columns (GE Healthcare). The 6 × His SUMO tag was removed by cleavage with the Sumo protease Ulp1 at a protease: protein ratio of 1:1000), and the tag was separated from the PPARγ LBD by a second pass through the Nickel column. PPARγ monomer was further purified by gel filtration (HiLoad 26/60 Superdex 200 (GE Healthcare)) with 20 mM of Tris (pH 8.0), 100 mM of NaCl, and 5 mM of DTT as buffer. The PPARγ LBD protein was complexed with PGC1α1 peptide (AEEPSLLKKLLLAPA) at a 1:1.2 molar ratio and with compound at a 1:5 molar ratio and then filter-concentrated to 10 mg/mL.

### Cell-based transactivation assay

Cos-7 cells from ATCC were grown to 70% confluence in DMEM containing 10% fetal bovine serum (FBS). For assessing full-length PPAR receptors, Cos-7 cells were transiently co-transfected with 100 ng plasmid containing the luciferase gene under the control of three tandem PPAR response elements (PPRE × 3 TK-luciferase) and 50 ng of full-length hPPARγ expression plasmid using lipofectamine 2000 (Invitrogen) along with 10 ng of Renilla luciferase expression plasmid for standardization. 24 hrs after transfection the cells were treated with the Rosi and compounds at the indicated concentration for 24 h. Luciferase activity was determined with the reporter luciferase assay kit (Promega) according to the manufacturer’s instructions using an Envision luminometer (Perkin–Elmer). Luciferase activity was normalized to Renilla activity. Each condition was performed with n ≥ 3 for each experiment. As a control, the activity was measured in the presence of vehicle (DMSO).

### LanthaScreen

The GST-PPARγ LBD was labeled with a terbium-linked antibody and incubated with a fluorescent small molecule pan-PPAR ligand (Fluormone™ Pan-PPAR Green). Excitation of terbium fluorescence at 340 nm results in a time-resolved fluorescence energy transfer (TR-FRET) to bound Fluormone with an emission at 520 nm. Agonist binding was determined as decrease in FRET (ratio of emission at 520 nm to emission from terbium fluorescence at 495 nm) due to the displacement of the fluorescent tracer from the ligand binding domain upon agonist binding. The inhibition constant (*k*_*i*_) for competitor was calculated by applying the Cheng-Prusoff equation as following:


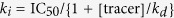


where IC_50_ is the concentration of competitor that produces 50% displacement of the tracer, [tracer] is the concentration of Fluormone™ Pan-PPAR Green used in the assay (5 nM), and *k*_*d*_ is the binding constant of Fluormone™ Pan-PPAR Green to PPAR γ-LBD (determined to be 2.8 ± 0.8 nM, (n = 3, average ± SD)).

### Differentiation

The adipocyte differentiation assay was performed with NIH 3T3-L1 preadipocytes obtained from ATCC. Preadipocytes were maintained in DMEM containing 10% FBS and antibiotics. Cells were induced by treating with 1 μM of dexamethasone, 0.5 mM of isobutylmethylxanthine, and 850 nM of insulin for 48 h and cells were switched to maintenance medium containing 850 nM of insulin for 6 days. The concentration of VSP-51 was used at 1 μM. Medium with DMSO was used as a negative control and 1 μM of Rosi was used as a positive control for the assay. Media were changed every two days. Lipid accumulation in the cells was detected by Oil Red O staining. The images were analyzed using ImageJ (National Institutes of Health).

### Gene expression analysis

For real-time PCR analysis, 1–2 μg total RNA was reverse-transcribed using the SuperScript cDNA Reverse Transcription Kit (Invitrogen) with SYBR Green PCR Master Mix (Invitrogen) and gene specific primers (Table S1) using an Roche Light Cycler 480 machine. The relative expression of mRNA was determined after normalization to β-actin level using the ΔΔ-Ct method.

### Crystallization, data collection and structure determination

The PPARγ/PGC-1α/VSP-51 crystals were grown at 25 °C in sitting drops containing 0.1 μL of the protein complex and 0.1 μL of well solution containing 0.1 M of tri-sodium citrate (pH 5.5), 20% w/v PEG 3350. Crystals were directly frozen in liquid nitrogen for data collection.

### Data collection and structure determination

The crystals formed in the C222_1_ space group (Table 1). The datasets were collected with a MAR225 CCD detector at the ID line of sector 21 of the Advanced Photon Source at Argonne National Laboratory (Argonne, IL). The data was indexed and scaled with HKL2000 package^49^ to 1.93 Å. The CCP4 program PHASER was used for molecular replacement (http://www.ccp4.ac.uk), with the PPARγ-Rosi structure (PBD code: 1FM6)^28^ as a search model. The initial model was manually built and refined with the PHENIX program package^50^. All figures were prepared using PyMOL (DeLano Scientific, San Carlos, CA, http://www.pymol.org).

## Additional Information

**How to cite this article**: Yi, W. *et al*. Identification of a novel selective PPARγ ligand with a unique binding mode and improved therapeutic profile *in vitro. Sci. Rep.* **7**, 41487; doi: 10.1038/srep41487 (2017).

**Publisher's note:** Springer Nature remains neutral with regard to jurisdictional claims in published maps and institutional affiliations.

## Supplementary Material

Supplementary Information

## Figures and Tables

**Table 1:** Data collection and refinement statistics.

	PPARγ LBD/compound 51
	Data collection
Space group	C222_1_
Resolution, Å	50–1.92 (1.95–1.92)
Cell parameters a, b, c, Å	56.9, 88.5, 122.2
α, β, γ, °	90, 90, 90
Total reflections	191088
Unique reflections	23627
Rsym	0.08(0.79)
I/σ	26.6 (2.3)
Completeness, %	99.9 (99.6)
Redundancy	8.1 (7.3)
Structure determination and refinement	
Resolution, Å	37.7-1.93
No. of reflections	23586
No. of residues	281
No. of solvent molecules	165
No. of non-H atoms	2441
Rwork	20.5 (29.7)
Rfree	23.4 (35.3)
RMSD bonds, Å	0.008
RMSD angles, °	1.157
Average B factor, Å^2^	41.1
Ramachandran	
Outliers, %	0.00
Favored, %	98.56
Clash score	2.17
Rotamer outliers, %	0.0
