# Supplementary material for: Identification of a novel selective PPARγ ligand with a unique binding mode and improved therapeutic profile in vitro
Source: Sci Rep. 2017 Jan 27;7:41487. doi: 10.1038/srep41487 (PMC5270246; doi:10.1038/srep41487)

**Supplementary Information**

**Title of manuscript:**

Identification of a novel selective PPARγ ligand with a unique binding mode and improved therapeutic profile in vitro

**Authors:**

Wei Yi, Jingjing Shi, Guanguan Zhao, X. Edward Zhou, [Kelly Suino-Powell](https://www.researchgate.net/profile/Kelly_Suino-Powell), [Karsten Melcher](https://www.researchgate.net/researcher/16162439_Karsten_Melcher), and [H. Eric Xu](https://www.researchgate.net/profile/Eric_Xu10)

**Supplementary information includes:**

Supplementary Table 1 and Figs. S1-S3

**Supplementary Table 1.** Primer sequences used for qPCR.

| **Gene** | **Forward primer** | **Reverse primer** |
| --- | --- | --- |
| C/EBPα | CAAGAACAGCAACGAGTACCG | GTCACTGGTCAACTCCAGCAC |
| aP2 | AAGGTGAAGAGCATCATAACCCT | TCACGCCTTTCATAACACATTCC |
| CD36 | AAGCTATTGCGACATGATT | GATCCGAACACAGCGTAGAT |
| FASN | GCTGGCATTCGTGATGGAGTCGT | AGGCCACCAGTGATGATGTAACTCT |
| LPL | GGGAGTTTGGCTCCAGAGTTT | TGTGTCTTCAGGGGTCCTTAG |
| PPARγ | GCATGGTGCCTTCGCTGA | TGGCATCTCTGTGTCAACCATG |
| Adiponectin | TGTTCCTCTTAATCCTGCCCA | CCAACCTGCACAAGTTCCCTT |
| PTP1B | CACCCTATCCCTGTAAATC | CCTTCACCAGTCTTGCTT |
| SOCS3 | GTGAAGAGGCAGTAGCA | TCTCCTAGCCCCACATAG |

**Supplementary Figure 1.** Structure-based design of novel indoles as PPARγ ligands.

**Supplementary Figure 2.** Comparison of the PPARγ-binding modes of VSP-51 and telmisartan. (A) Structural overlay of VSP-51 (pink) and telmisartan (grey) in the PPARγ ligand-binding pocket (magenta: Telmisartan–bound structure, green: VSP-51-bound structure). (B) Interactions of telmisartan (grey) with key PPARγ residues shown in magenta. (C) Interactions of VSP-51 (pink) with key PPARγ residues shown in green. Dashed lines indicate hydrogen bonds and arrows indicate significant rotamer changes of receptor residues caused by the bulky groups of telmisartan in its complex structure.


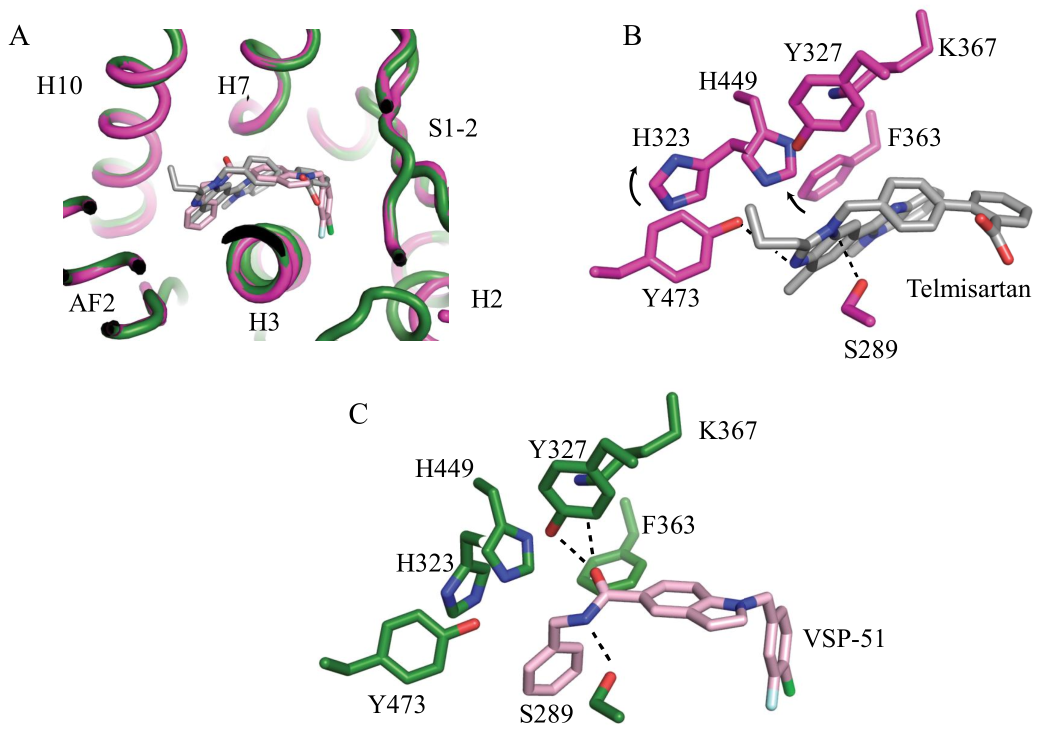


**Supplementary Figure 3.** Comparison of SR1663, SR1664, and VSP-51 binding modes. (A) Structural overlay of SR1663 (yellow) and SR1664 (blue) in the PPARγ ligand-binding pocket. The binding modes of SR1664 (B) and SR1663 (C) are also shown separately. (D, E) Binding mode of VSP-51 (green, D) and its overlay SR1663 (yellow, E). Dashed lines indicate hydrogen bonds between receptor residues and ligands.


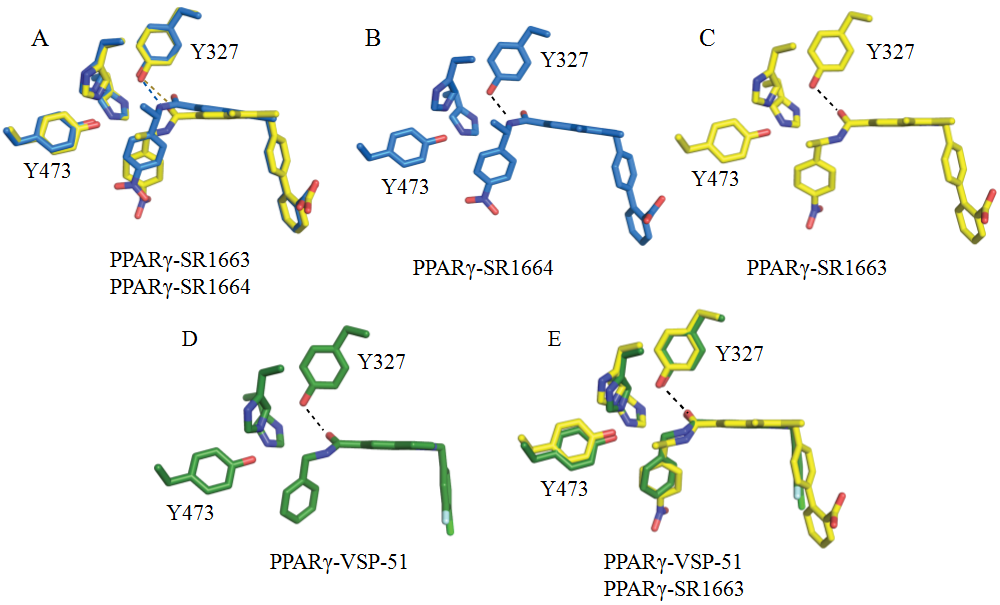

Supplement: Supplementary Information [file srep41487-s1.doc]
